# *Trigonellae* Semen Enhances Sperm Motility and the Expression of the Cation Sperm Channel Proteins in Mouse Testes

**DOI:** 10.1155/2015/817324

**Published:** 2015-10-11

**Authors:** Do Rim Kim, Hyu Young Kim, Ha Young Kim, Mun Seog Chang, Seong Kyu Park

**Affiliations:** Department of Prescriptionology, College of Korean Medicine, Kyung Hee University, Seoul 130-701, Republic of Korea

## Abstract

Genetic defects during spermatogenesis can lead to a reduction in sperm motility and cause male infertility. The cation channels of sperm (CatSper) play a role in the regulation of hyperactivated sperm motility in mouse testes. The effect of *Trigonellae* Semen (TS) on the male reproductive system and CatSper protein in mouse testes during spermatogenesis was examined. C57BL/c mice were divided into the following five groups: normal, cyclophosphamide- (CP-) only treated (control group), and three groups treated with varying concentrations of TS with CP (100, 500, and 1000 mg/kg TS and 100 mg/kg CP). Real-time PCR, western blot analysis, and a testosterone immunoassay were performed to assess CatSper protein levels in the five groups. Additionally, sperm cell counts and motility were examined. Results indicate that sperm motility and sperm counts increased in the TS treated groups in a dose-dependent manner (*p* < 0.01). CatSper levels were also significantly higher in the TS treated groups compared to that of the control group (*p* < 0.001). Therefore, TS treatment could enhance sperm function by promoting spermatogenesis and the expression of CatSper proteins in mouse testes.

## 1. Introduction

Spermatogenesis is a complex process of male germ cell proliferation, differentiation, and maturation from diploid spermatogonia to haploid spermatozoa in the seminiferous tubules of the testes [1]. The biological process of sperm production is regulated hormonally through feedback mechanisms using Leydig cells to promote testosterone production and cell signaling such as a Sertoli and Leydig cell [2]. This paracrine and endocrine regulation of germ cell development requires spermatogenic stage- and cell-specific gene expression [3]. It has been estimated that over 2000 proteins are involved in the specialized regulation of spermatogenesis [4, 5]. Genetic disorders such as chromosomal abnormalities or single gene mutations can impair spermatogenesis or reduce sperm cell function and can result in male infertility [5–8]. The development of assisted reproductive technologies can overcome male infertility; however, the genetic defects may still be passed to the male's offspring. Therefore, it is necessary to investigate the effects of controlled drugs for testis-specific gene expression or identify novel genetic biomarkers of normal spermatogenesis.

Cation channels of sperm (CatSper) are composed of four separate pore-forming *α* subunits (CatSper 1–4) and auxiliary subunits (*β*, *γ*, and *δ*) [9]. CatSper 1–4 transcripts are differentially expressed at the time of spermatogenesis in the testes and are localized to the principal piece of the sperm tail. The expression of CatSper 1, 3, and 4 is restricted to spermatids, whereas CatSper 2 is transcribed in the early stages of spermatogenesis (pachytene spermatocytes) [10, 11]. CatSper channels are also named as Ca^2+^ ion channels that mediate sperm hyperactivation [12]. CatSper gene expression levels were significantly lower in males with low sperm motility than males with normal fertility [13]. A recent study reported that male mice with CatSper 3 and 4 genes knocked out displayed infertility due to a lack of hyperactivated sperm motility, despite the initial presence of normal sperm counts and motility [14].

*Trigonellae* Semen (TS) is derived from the dry and ripe seeds of* Trigonella foenum-graecum *L., which belongs to Leguminosae family. It has commonly been used in medicine to tonify the kidneys and provide pain relief. Additionally, it has also been reported to have antidiabetic activity [15, 16], anticholesterolemic effects [17, 18], a curative gastric antiulcer action [19], and antibacterial [20], anthelmintic [21], and antinociceptive effects [22]. The broad biological and pharmacological actions of TS are attributed to the variety of its constituents, namely, steroids, *n*-compounds, polyphenolic substances, volatile constituents, and amino acids [23–25]. TS is a medicinal herb used for the treatment of infertility and impotence in Korean medicine. However, the effect of TS on spermatogenesis-related gene expression and levels of encoded protein in mouse testes have yet to be determined.

This study investigated the effects of the TS extract on spermatogenesis and CatSper gene expression in mice using cyclophosphamide (CP) to induce testicular toxicity. Sperm count and motility, serum testosterone levels, and CatSper protein levels were assessed to evaluate the effects of TS on spermatogenesis.

## 2. Materials and Methods

### 2.1. Preparation of* Trigonellae* Semen Extract

TS, the seed of* T. foenum-graecum*, was purchased from Wonkwang Herbal Drug Co. Ltd. (Korea). Three hundred grams of dried TS was boiled in 6 L of water for 2 h at 100°C. The suspension was filtered and concentrated under reduced pressure. The filtrate was then lyophilized and yielded 60.27 g (20.09%) of powder, which was stored at 4°C.

### 2.2. Animals and Experimental Protocol

Five-week-old male C57BL/c mice were purchased from SLC Inc. (Japan). The animals were housed in a specific pathogen-free environment with a 12 h light : dark cycle at the Center for Laboratory Animal Care and Use at Kyung Hee University. Animal care and experimental procedures conformed to the “Guide for the Care and Use of Laboratory Animals” (Department of Health, Education, and Welfare, NIH publication # 78-23, 1996). Animals had free access to standard rodent pellets (Purina, Korea) and water.

After 7 days of adaptation to the environment, the mice were divided into the following five groups: normal group (N: vehicle-treated, *n* = 8), control group (C: cyclophosphamide (CP) 100 mg/kg, i.p. only treated, *n* = 10), and groups treated with three concentrations of TS and CP (CP + TS: 100, 500, and 1000 mg/kg TS and CP 100 mg/kg, *n* = 10/group). The animals were weighed weekly to adjust the gavage volume and to monitor their general health. At the end of the treatment period, the mice were anesthetized with urethane (100 mg/kg, i.p.). Serum was separated from the whole blood collected by cardiac puncture and stored in the deep-freezer until required for quantitative serum testosterone analysis. The testes were removed and cleared of the adhering tissues and weighed. The epididymis was removed for use for sperm analysis. The testes samples were frozen for use in real-time PCR and western blotting assays.

### 2.3. Sperm Cell Count and Motility

To obtain the sperm cell count, the entire epididymis from the mouse was minced in a sperm washing medium and incubated for 30 min at 37°C. Total epididymal sperm cell counts and motility were evaluated using the Computer Assisted Semen Analysis (CASA) system (Hamilton Thorne, USA). Sperm cells were scored as motile if any movement was detected.

### 2.4. Testosterone Immunoassay

Testosterone levels were determined using serum samples and a testosterone immunoassay kit following the manufacturer's protocols (R&D systems, USA). The samples were tested in triplicate and compared to two testosterone control standards.

### 2.5. Real-Time PCR Analysis

Total RNA was extracted from each mouse. cDNA synthesis was performed using 5 *μ*g of total RNA with MMLV reverse transcriptase and oligo-dT primers for 1 h at 42°C. Real-time PCR was performed using a total reaction volume of 20 *μ*L containing the following: 2 *μ*L (200 ng) of cDNA, 10 *μ*L of PCR master mix, 1 *μ*L of each TaqMan probe, and 7 *μ*L of diethyl pyrocarbonate-treated water. The samples were tested using the Applied Biosystems StepOnePlus Real-Time PCR System (Applied Biosystems, USA). The program parameters used were a 50°C hold for 2 min and a 95°C hold for 10 min, followed by 40 cycles of denaturation at 95°C for 15 s and annealing at 60°C for 60 s. The primers and probes for the CREM gene and the housekeeping gene GAPDH were predesigned by Applied Biosystems. As a control for the input amount, each cDNA sample was also amplified using the predesigned primers and probes (assay ID: Mm00460530_m1 (CatSper 1), Mm00467632_m1 (CatSper 2), Mm00712792_m1 (CatSper 3), Mm01190761_m1 (CatSper 4), and Mm99999915_g1 (GAPDH), Applied Biosystems, USA). Samples were amplified with GAPDH primers for determination of the initial relative quantity of cDNA in each sample, and then all PCR products were normalized to that amount. Nontemplate controls were used for each run. Samples were amplified in triplicate, the averages were calculated, and the differences in the relative quantity were evaluated using the StepOne Software v. 2.1 (Applied Biosystems, USA).

### 2.6. Western Blot Analysis

Proteins from homogenized testes were separated using a nuclear extraction kit following a modification of the manufacturer's protocol (Active & Motif, USA). SDS-PAGE and western blotting were performed as described previously [26]. Samples for protein extraction were half of the same testes used for RNA extractions. Equivalent amount (50 *μ*g) of protein extracts was separated in 10% Tris-glycine gels by SDS-PAGE and transferred to nitrocellulose membranes using 25 mM Tris and 250 mM glycine buffer containing 20% methanol, pH 8.3. Transfer was performed at a constant voltage of 120 mA for 1 h. After transfer, the membranes were blocked in phosphate-buffered saline (PBS) containing 0.05% Tween (PBS-T) with 5% skim milk for 2 h at room temperature and incubated with the primary antibodies (1 : 1000) for CatSepr 1 (sc-21180), CatSper 2 (sc-98539), CatSper 3 (sc-98818), and CatSper 4 (sc-83126) in PBS-T overnight at 4°C. Following overnight incubation, the membranes were rinsed with 1x PBS three times and incubated with conjugated goat anti-rabbit IgG for 1 h at room temperature, followed by three additional washes with 1x PBS.

### 2.7. Statistical Analysis

The results were expressed as the means ± standard deviation (SD). Differences between the groups were assessed by one-way ANOVA using the SPSS software package for Windows. *p* values of <0.001, <0.01, and <0.05 were considered to indicate statistical significance.

## 3. Results

### 3.1. Body and Testes Weights after TS Treatment

The body and testes weights were measured on the day following full treatments (Table 1). The body weights and absolute and relative weights of testes in the CP and TS treated groups (100, 500, and 1000 mg/kg) were significantly increased compared to the control group (treated with CP only).

### 3.2. Sperm Counts and Motilities

**Figure 1:**
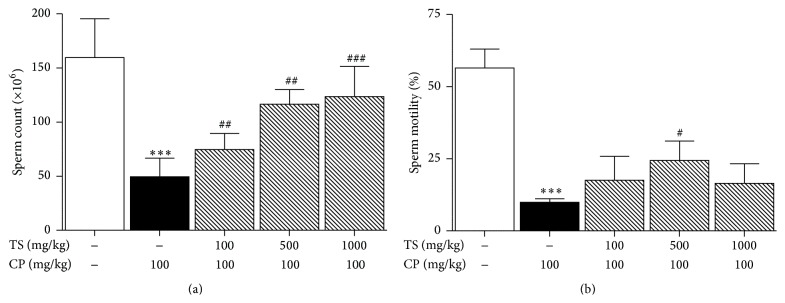
Effect of TS and CP on sperm count and motility. Normal, vehicle-treated group. Control, CP (100 mg/kg/week, i.p. 5 weeks) treated group. The CP and TS groups received CP (100 mg/kg) and TS (100, 500, and 1000 mg/kg, p.o., 5 weeks). (a) Sperm cell count and (b) sperm motility. Each column represents the mean ± SD (*n* = 5). *∗* means significantly different from the normal value (_ _^*∗∗∗*^ *p* < 0.001). # indicates that the mean is significantly different from the control value (_ _^#^ *p* < 0.05, _ _^##^ *p* < 0.01, and _ _^###^ *p* < 0.001).

The epididymal sperm counts of the CP and TS treated groups (100, 500, and 1000 mg/kg) were significantly higher than that of the control group (74.56 ± 14.91, 116.58 ± 13.47, and 123.44 ± 28.02 × 10^6^, resp.; *p* < 0.01, Figure 1(a)). Additionally, sperm cell motility in the CP and TS groups (100, 500, and 1000 mg/kg) was greater than that in the control group (17.56 ± 8.24, 24.41 ± 6.69, and 16.49 ± 6.75%, resp.; *p* < 0.05, Figure 1(b)). Sperm velocity parameters such as average path velocity (VAP, *μ*m/s), straight line velocity (VSL, *μ*m/s), curvilinear velocity (VCL, *μ*m/s), and amplitude of lateral head displacement (ALH) of control group have significantly decreased compared to normal group. CP and TS groups were increased compared to control group (Table 2).

### 3.3. Effect of TS on Serum Testosterone Levels

**Figure 2:**
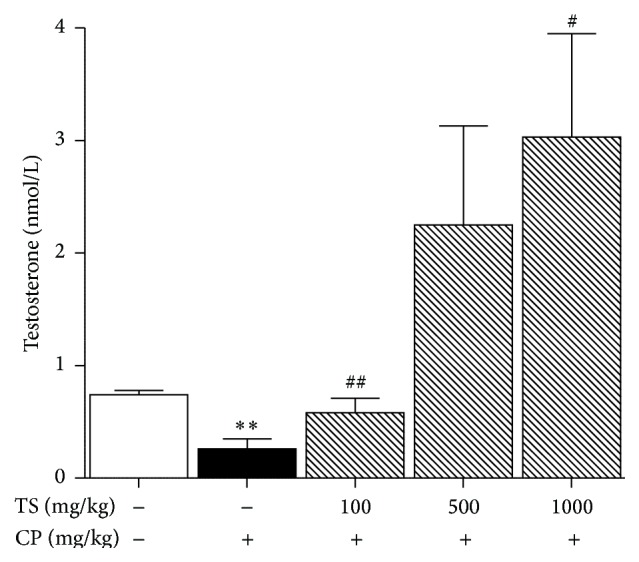
Effect of TS on serum testosterone levels in CP treated mice. Normal, vehicle-treated group. Control, CP (100 mg/kg/week, i.p. 5 weeks) treated group. The CP and TS groups received CP (100 mg/kg) and TS (100, 500, and 1000 mg/kg, p.o., 5 weeks). Each column represents the mean ± SD (*n* = 5). *∗* indicates that the mean is significantly different from the normal value (_ _^*∗*^ *p* < 0.05, _ _^*∗∗*^ *p* < 0.01). # indicates that the mean is significantly different from the control value (_ _^##^ *p* < 0.01).

The serum testosterone levels of mice treated with CP significantly decreased by 65% when compared to the normal group (0.74 ± 0.04 versus 0.26 ± 0.09 nmol/L, *p* < 0.01). Furthermore, it was observed that samples treated with CP and TS also increased as the concentration of TS increased (0.26 ± 0.09 versus 0.58 ± 0.13, 2.25 ± 0.88, and 3.03 ± 0.92 nmol/L, respectively (Figure 2)).

### 3.4. Effect of TS on CatSper mRNA Levels in Mouse Testes

**Figure 3:**
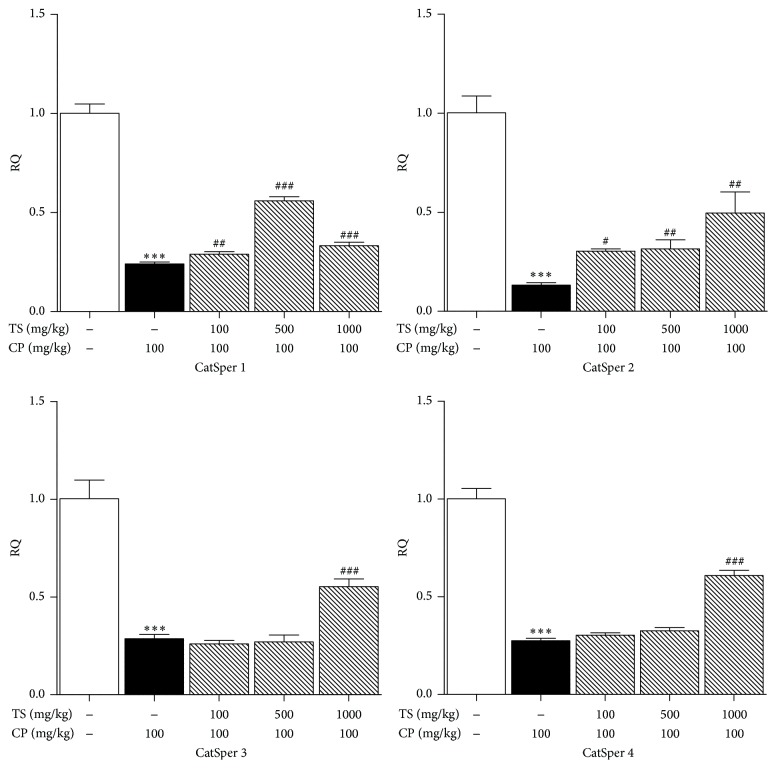
Real-time PCR analysis of CatSper 1–4 gene expression in TS and CP treated mice testes. Normal, vehicle-treated group. Control, CP (100 mg/kg/week, i.p. 5 weeks) treated group. The CP and TS groups received CP (100 mg/kg) and TS (100, 500, and 1000 mg/kg, p.o., 5 weeks). The level of CatSper mRNA was normalized to the GAPDH reference signal. RQ refers to the relative quantity of gene expression. Each column represents the mean ± SD (*n* = 3). *∗* indicates that the mean is significantly different from the normal value (_ _^*∗∗∗*^ *p* < 0.001). # indicates that the mean is significantly different from the control value (_ _^#^ *p* < 0.05, _ _^###^ *p* < 0.001).

To determine the effect of TS on CatSper 1–4 mRNA levels in mouse testes, CatSper 1–4 mRNA levels were analyzed using real-time PCR. The normal group is regarded as the standard value (relative quantity; RQ = 1). The relative quantity for the control groups (CatSper 1–4) decreased significantly (RQ = 0.24, 0.13, 0.28, and 0.27, *p* < 0.001, resp.). CatSper 1 mRNA levels in mouse testes treated with CP and 500 mg/kg TS increased significantly, as did CatSper 2, 3, and 4 mRNA levels in mouse testes treated with CP and 1000 mg/kg TS, compared to the levels in the control group (Figure 3).

### 3.5. Effect of TS on CatSper Protein Levels in Mouse Testes

**Figure 4:**
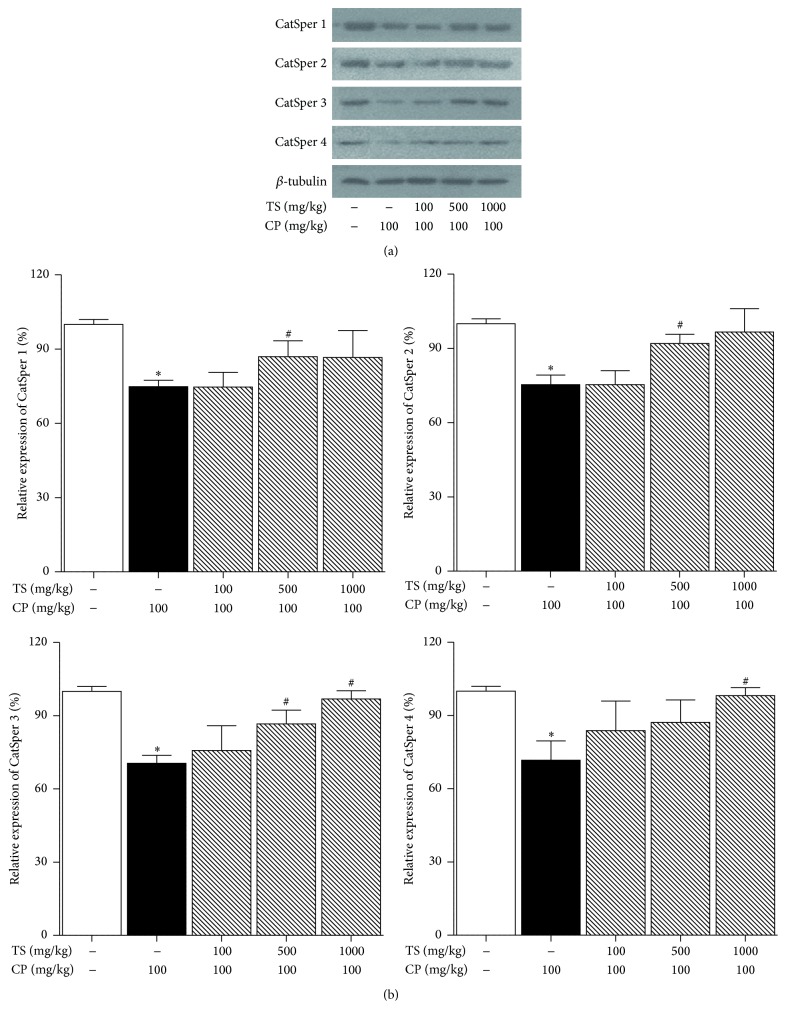
Effect of TS on CatSper 1–4 protein levels in TS and CP treated mice testes. Normal, vehicle-treated group. Control, CP (100 mg/kg/week, i.p. 5 weeks) treated group. The CP and TS groups received CP (100 mg/kg) and TS (100, 500, and 1000 mg/kg, p.o., 5 weeks). *β*-tubulin was used as an internal control. Each column represents the mean ± SD (*n* = 3). *∗* indicates that the mean is significantly different from the normal value (_ _^*∗*^ *p* < 0.05).

Samples treated with CP showed a decrease in CatSper protein levels (*p* < 0.05). However, the levels were recovered in samples treated with both CP and TS (Figure 4).

## 4. Discussion and Conclusions

The aim of this study was to investigate the effect of* Trigonellae* Semen on the male reproductive system and CatSper expressions in mouse testes during spermatogenesis. As an anticancer chemotherapeutic drug typically used as an immunosuppressive agent for organ transplantation, systemic lupus erythematosus, multiple sclerosis, and other benign diseases [27], cyclophosphamide (CP) was used to induce reproductive toxicity in the experimental rodents. The bioactivated metabolites of CP cause cross-linking of the DNA strands, preventing cell division and causing damaging to the testes [28].

The testes weights were measured the day following administration of the prescribed treatment. The absolute and relative testes weights in the CP treated group significantly decreased compared to the normal group. In contrast, the absolute and relative weights of testes in the CP and TS treated groups increased.

Sperm cell analysis and histopathological examination of the testes are the most effective methods for the detection of male reproductive disorders [29]. Sperm cell count and motility were estimated after isolation of sperm from mouse epididymis. The epididymal sperm count and motility of the mice treated with CP were significantly decreased compared to the control group. In contrast, the CP and TS treated groups showed an increased sperm count compared to the CP-only treated group. Notably, the number of sperm cells was significantly higher than that in the CP-only treated group. In results about sperm motion parameters, VAP, VSL, VCL, and ALH of control group have significantly decreased compared to normal group, while CP and TS treated groups were increased compared to control group. Similar to the testicular weights, this increase occurred in a dose-dependent manner.

The production of a normal number of spermatozoa is highly dependent on the regulation of gene expression in the germ cells, paracrine signaling and hormonal control of germ cell proliferation, and differentiation. The germ cells are supported structurally, nutritionally, and functionally by the Sertoli cells. The Leydig cells are adjacent to the Sertoli cells on the nonluminal side of the seminiferous tubules and produce testosterone [30]. The pituitary gonadotropin-luteinizing hormone (LH) stimulates testosterone synthesis in the Leydig cells. As a primary regulator of spermatogenesis, testosterone, together with follicle-stimulating hormone (FSH), causes Sertoli cells to secrete the growth factors and peptides required for germ cell differentiation [31, 32]. It is, therefore, imperative to maintain the level of testosterone secretion by Leydig cells to ensure proper spermatogenesis [33]. In this study, the serum testosterone levels in the CP and TS treated groups increased in a dose-dependent manner.

Sperm motility is a significant indicator of fertilization capability. Spermatozoa differentiate to mature spermatozoon by testis-specific gene regulation during spermatogenesis [34]. For successful fertilization, hyperactivation, a type of sperm motility, is required. The hyperactivated sperm swim vigorously and generate enough force to penetrate the cumulus cells and zona pellucida of the egg cell during fertilization [35]. Hyperactivated sperm motility is regulated by the intracellular Ca^2+^ concentration. Ca^2+^ influx through CatSper channels induces hyperactivated sperm motility [36].

To investigate the effects of* Trigonellae* Semen on CatSper expression, real-time PCR and western blotting assays were performed. CatSper 1, 2, 3, and 4 protein levels decreased due to reproductive toxicity caused by CP. However, CatSper mRNA levels of mouse testes treated with CP and TS were increased compared to that in the CP-only treated group (the control group). The western blot assay showed that the protein levels in the CP and TS treated groups were higher than those in the control group. These results indicate that TS stimulates the hyperactivity of sperm motility through activation of CatSper channels.

In conclusion,* Trigonellae* Semen has a protective effect on CP-induced infertile male mice by enhancing testosterone secretion and increasing sperm count and sperm motility. In addition,* Trigonellae* Semen upregulated CatSper mRNA and protein levels, thus protecting sperm motility against the damage caused by CP. Our results suggest that TS could be of help in subjects treated with CP in other diseases.

## Figures and Tables

**Table 1:** Body and testicular weights following TS treatment.

Group^(1)^	Body weight (g)	Absolute testes weight (g)	Relative testes weight (%)
Normal	25.58 ± 2.81^(2)^	0.094 ± 0.004	0.37 ± 0.039
Control	20.93 ± 0.95	0.031 ± 0.002^*∗∗∗*^	0.15 ± 0.004^*∗∗*^
CP + TS 100	22.03 ± 1.35	0.039 ± 0.003^#^	0.17 ± 0.008^#^
CP + TS 500	23.20 ± 3.23	0.045 ± 0.006^#^	0.19 ± 0.002^###^
CP + TS 1000	22.90 ± 1.44	0.046 ± 0.005^##^	0.20 ± 0.012^##^

^(1)^Normal: vehicle-treated group.

Control: cyclophosphamide-only treated group (100 mg/kg, i.p., 5 weeks).

CP + TS: CP (100 mg/kg, i.p., 5 weeks) and TS (100, 500, and 1000 mg/kg/day, p.o., 5 weeks) treated group.

^(2)^Values are the means ± SD (*n* = 8).

*∗* indicates that the mean is significantly different from the normal value (^*∗∗*^ *p* < 0.01, ^*∗∗∗*^ *p* < 0.001).

# indicates that the mean is significantly different from the control value (^#^ *p* < 0.05, ^##^ *p* < 0.01, and ^###^ *p* < 0.001).

**Table 2:** Sperm parameter with TS.

Groups^(1)^	VAP (*μ*m/s)^(2)^			VSL (*μ*m/s)^(2)^			VCL (*μ*m/s)^(2)^			ALH (*μ*m/s)^(2)^		
	Mean	±SD	*p*	Mean	±SD	*t*-test	Mean	±SD	*p*	Mean	±SD	*p*
Normal	63.23	4.22	—	45.88	3.18	—	110.10	7.68	—	7.45	0.35	—
Control	44.06	5.09	0.01	30.04	2.98	0.003	77.52	11.19	0.011	4.76	0.73	0.002
CP/TS 100 mg/kg	54.99	3.24	0.014	39.89	3.00	0.01	91.82	5.05	0.1	5.92	0.83	0.1
CP/TS 500 mg/kg	51.61	2.98	0.03	35.73	1.68	0.02	90.09	5.50	0.03	6.81	0.50	0.0011
CP/TS 1000 mg/kg	54.93	4.01	0.001	38.97	3.42	0.0003	93.27	6.64	0.01	6.52	0.24	0.01

^(1)^Normal: vehicle-treated group. Control: cyclophosphamide (CP) (100 mg/kg, i.p., 5 weeks) treated group. CP/TS: cyclophosphamide (100 mg/kg, i.p., 5 weeks), and *Trigonellae* Semen (100, 500, and 1000 mg/kg/day, p.o., 5 weeks) treated group.

^(2)^VAP, average path velocity (*μ*m/s); VSL, straight line velocity (*μ*m/s); VCL, curvilinear velocity (*μ*m/s); ALH, amplitude of lateral head displacement (*μ*m/s).
